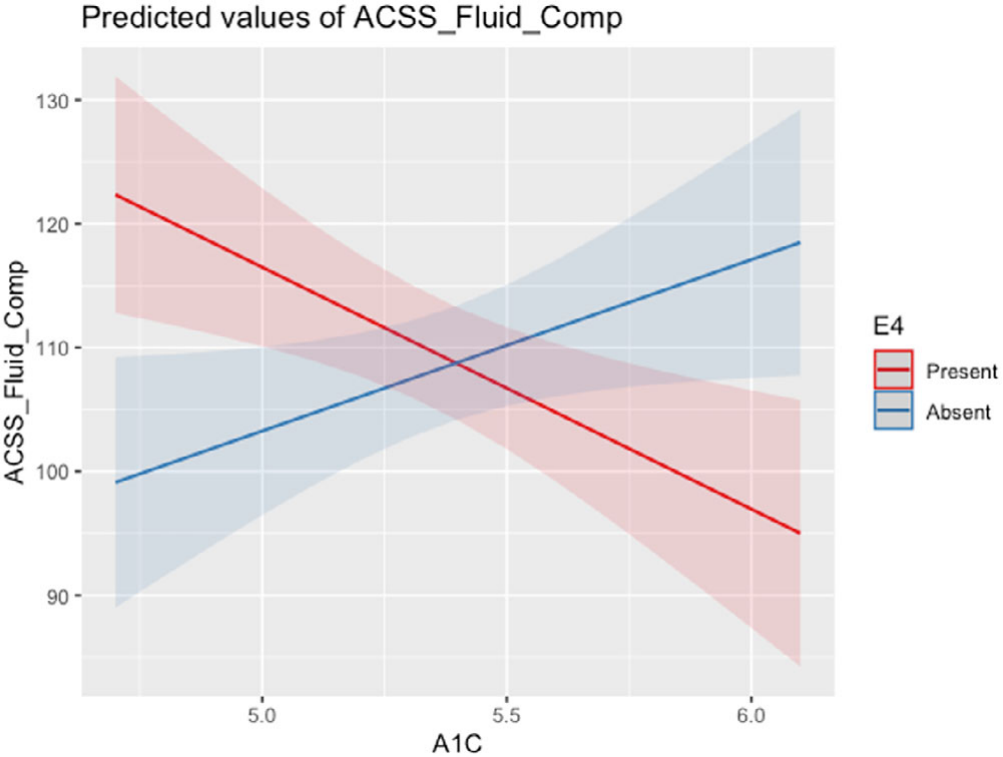# Exploring the Relationship Between HbA1c, APOE4 Status, and Fluid Cognitive Performance in Healthy Older Adults

**DOI:** 10.1002/alz70860_105306

**Published:** 2025-12-23

**Authors:** Jacob F Reusch, Kristen M Farris, Jasroop K Miglani, Angela J Hanson

**Affiliations:** ^1^ University of Washington School of Medicine, Seattle, WA, USA

## Abstract

**Background:**

Several ongoing clinical trials are investigating the potential protective effects of diabetes medications in dementia prevention. While diabetes is a known risk factor for Alzheimer's disease (AD), less is known about the relationship between diabetes risk and those with the APOE4 genotype. We examined how metabolic factors are related to performance and cognitive outcomes using the NIH Toolbox Cognition Battery (NIHTB‐CB) as part of two high‐fat meal intervention studies in older adults without diabetes.

**Method:**

139 older adults (age 66.28 ± 7.06, 77 females) underwent metabolic screening, including fasting labs and vitals. On a separate day, participants completed the NIHTB‐CB following a high‐fat intervention (either a high fat meal or high fat drink). General linear models and ANOVAs in R were used to examine the relationship between metabolic factors, APOE4 status, and cognition. Further exploratory correlations were done in SAS. All analyses were adjusted for age and education.

**Result:**

Hemoglobin A1C (HbA1c) individually predicted test performance on Flanker (*p* = 0.037), List Sorting (*p* = 0.048), Dimensional Change Card Sort (*p* = 0.003), Pattern Comparison (*p* = 0.042), Fluid Composite (*p* = 0.004), and Total Composite (*p* = 0.002). A significant interaction was found between HbA1c and E4 status on test performance on Flanker (*p* = 0.007), List Sorting (*p* = 0.010), Dimensional Change Card Sort (*p* = 0.004), Pattern Comparison (*p* = 0.014), Fluid Composite (*p* = 0.001), and Total Composite (*p* = 0.001). Specifically, higher HbA1c was predictive of better fluid cognitive performance in non‐E4 carriers (*p* = 0.038, Figure), while the opposite was true for E4 carriers (*p* = 0.001). ANOVA analysis showed no statistically significant differences in cognitive performance or HbA1c levels between E4 groups and confirmed the E4*HbA1C interaction for all fluid cognitive measures. No relationship was found for other metabolic factors including blood pressure, LDL, and total cholesterol.

**Conclusion:**

In cognitively normal older adults without diabetes, E4 status had an interactive effect with HbA1c. We found that higher A1c predicted better cognitive performance in E4 non‐carriers, but worse cognitive performance in E4 carriers. Understanding the interplay between metabolic factors, E4 status, and cognition can provide insight into AD risk and prevention. This is critical given ongoing research involving diabetes medications such as GLP‐1s and their potential role in AD prevention.